# Solution Structure of the Cuz1 AN1 Zinc Finger Domain: An Exposed LDFLP Motif Defines a Subfamily of AN1 Proteins

**DOI:** 10.1371/journal.pone.0163660

**Published:** 2016-09-23

**Authors:** Zhen-Yu J. Sun, Meera K. Bhanu, Martin G. Allan, Haribabu Arthanari, Gerhard Wagner, John Hanna

**Affiliations:** 1 Department of Biological Chemistry and Molecular Physiology, Harvard Medical School, Boston, Massachusetts, United States of America; 2 Department of Pathology, Brigham and Women's Hospital and Harvard Medical School, Boston, Massachusetts, United States of America; George Washington University, UNITED STATES

## Abstract

Zinc binding domains are common and versatile protein structural motifs that mediate diverse cellular functions. Among the many structurally distinct families of zinc finger (ZnF) proteins, the AN1 domain remains poorly characterized. Cuz1 is one of two AN1 ZnF proteins in the yeast *S*. *cerevisiae*, and is a stress-inducible protein that functions in protein degradation through direct interaction with the proteasome and Cdc48. Here we report the solution structure of the Cuz1 AN1 ZnF which reveals a compact C6H2 zinc-coordinating domain that resembles a two-finger hand holding a tri-helical clamp. A central phenylalanine residue sits between the two zinc-coordinating centers. The position of this phenylalanine, just before the penultimate zinc-chelating cysteine, is strongly conserved from yeast to man. This phenylalanine shows an exceptionally slow ring-flipping rate which likely contributes to the high rigidity and stability of the AN1 domain. In addition to the zinc-chelating residues, sequence analysis of Cuz1 indicates a second highly evolutionarily conserved motif. This LDFLP motif is shared with three human proteins—Zfand1, AIRAP, and AIRAP-L—the latter two of which share similar cellular functions with Cuz1. The LDFLP motif, while embedded within the zinc finger domain, is surface exposed, largely uninvolved in zinc chelation, and not required for the overall fold of the domain. The LDFLP motif was dispensable for Cuz1's major known functions, proteasome- and Cdc48-binding. These results provide the first structural characterization of the AN1 zinc finger domain, and suggest that the LDFLP motif may define a sub-family of evolutionarily conserved AN1 zinc finger proteins.

## Introduction

Zinc finger domains are among the most common protein structural motifs in eukaryotes. Sequence analysis in humans predicts over 1,000 distinct zinc finger-containing proteins [1]. As a family, zinc finger proteins are extraordinarily diverse, but can be grouped into smaller families based on the recognition of distinct structural modes of zinc coordination [2]. The functions of zinc finger proteins are equally diverse. Although originally described as a DNA-binding domain, it is now appreciated that zinc finger domains can mediate interactions with RNA, other proteins, and lipids [1].

The AN1 domain represents a broadly distributed and evolutionarily conserved family of zinc finger proteins [3]. The name derives from the identification of mRNAs that were specifically localized to the animal pole of the *Xenopus laevis* egg [4–5]. The AN1 domain shows a characteristic Cys6His2 organization that coordinates two zinc ions, and is part of a larger structural motif known as the treble clef domain [3]. Among zinc finger domains, AN1 proteins are poorly understood at both structural and functional levels.

Budding yeast *S*. *cerevisiae* contain two AN1-domain proteins, Cuz1 and Tmc1 [6–7]. Recent studies indicate a role for Cuz1 in regulated protein degradation. In eukaryotes, such degradation is carried out primarily by the ubiquitin-proteasome system. Substrates fated for destruction in this pathway first acquire covalent modification by the small protein ubiquitin, which then serves as a targeting signal for the proteasome, a large multisubunit protease [8]. The proteasome binds the ubiquitin signal, unfolds the protein, and degrades it into small peptides while releasing ubiquitin for reuse. A large multifunctional ATPase complex centered around Cdc48 plays key roles in protein degradation, and is thought to act on ubiquitinated proteins upstream of the proteasome. Cuz1 interacts directly with both the proteasome and Cdc48, suggesting an important role for Cuz1 in protein degradation, although the precise molecular function of Cuz1 in this process remains unclear [6–7].

We have undertaken a structural and functional analysis of Cuz1's AN1 domain. This represents the first reported structure of the AN1 ZnF, and reveals a novel mode of zinc coordination. Within Cuz1's ZnF, we identify a second highly conserved motif which appears to be largely uninvolved in zinc coordination and dispensable for the overall fold of the domain. We propose that this LDFLP motif defines a sub-family of evolutionarily conserved AN1 ZnF proteins.

## Materials and Methods

### Plasmids and Strains

Several candidate expression plasmids for the Cuz1 (systematic name: Ynl155w) AN1 zinc finger domain were constructed and tested. Optimal yield and purity were obtained with plasmid pJH190. This pET45b-based plasmid encodes for Cuz1 amino acids 11–59 with an N-terminal 6x-Histidine tag for affinity purification. The GST-Cuz1 bacterial expression plasmid, pJH150, has been previously described [7]. Full length GST-Cuz1^LDFL→AAAA^ was prepared by site-directed mutagenesis of pJH150, resulting in pJH171. The same mutation was introduced into pJH190, resulting in pJH219. Plasmids were verified by sequencing. Yeast were cultured at 30°C in YPD or selective media, as appropriate. YPD medium consisted of 1% yeast extract, 2% Bacto-peptone, and 2% dextrose.

### Recombinant Protein Purification

For structural analysis of the AN1 zinc finger domain, pJH190 (or pJH219) was expressed in BL21 (DE3) and cultured in M9 minimal media supplemented with zinc sulfate (50 μM) and carbenicillin (50 μg/mL). Logarithmic phase cultures were induced with IPTG (1 mM) and grown overnight at 20°C. Lysis buffer was phosphate buffered saline (PBS) pH 7.4, supplemented with imidazole (10 mM) and protease inhibitors (Roche). Lysates were prepared by French press, clarified by centrifugation in a SS-34 rotor for 25 min at 16,000xg, and filtered through cheesecloth. Protein was purified by Ni-NTA affinity chromatography (Qiagen), washed with PBS supplemented with NaCl (100 mM) and imidazole (20 mM), and eluted with PBS supplemented with imidazole (400 mM). The eluate was desalted using a PD-10 column (GE Healthcare Life Sciences) and then applied to a centrifugal filter with a 30 kDa cutoff (Millipore) to remove high molecular weight contaminants. ^15^N-labeled NH_4_Cl and ^13^C-labeled glucose (Cambridge Isotope Laboratories) were used to generate ^15^N- and ^15^N/^13^C-labeled protein. Standard size exclusion chromatography for analysis was carried out with a Superdex 75 16/60 column (GE Healthcare Life Sciences).

Full length wild-type and mutant GST-Cuz1 proteins were prepared by standard glutathione sepharose affinity chromatography as previously described [7]. 12xHis-SUMO-Cdc48 was prepared by standard Ni-NTA affinity chromatography as previously described [7].

### NMR Analysis

Cuz1 ZnF protein samples for NMR analysis were buffer-exchanged to 5 mM Tris, 50 mM NaCl, 0.2 mM ZnCl_2_, 1 mM DTT, pH 7.5, with 10% D_2_O using centrifuge concentrators with a 3 kDa cutoff. Triple resonance experiments for backbone and sidechain assignments, as well as ^15^N and ^13^C edited 3D-NOESY experiments, were performed non-uniformly sampled on an Agilent dd2600 spectrometer at 25°C using a 0.7 mM ^15^N-^13^C labeled Cuz1 sample. 2D-NOESY and TOCSY data in D_2_O were acquired on a Bruker 750 spectrometer at 25°C using a 0.85 mM unlabeled Cuz1 sample. NMR data were processed using NMRPipe [9] and hmsIST software [10] and analyzed using the CARA software [11]. The backbone dihedral angle constraints were obtained using the TALOS+ [12] software based on assigned ^15^N/^13^C-chemical shift values. The manually assigned NOE distance constraints were first calibrated by default values and then by iteratively computed structure models. The zinc cluster constraints were modeled according to ZAFF (zinc amber force field) [13]. One hundred NMR structures were calculated using CYANA software [14], each with 100,000 simulated annealing steps, from which 15 lowest energy conformers with no NOE violations greater than 0.3Å and no angular violations greater than 5° were selected as a final ensemble. A highly mobile 27 residue N-terminal cloning artifact containing a 6xHis-tag and an enterokinase cleavage site (MAHHHHHHVGTGSNDDDDKSPDPNWEL) was assigned but not included in the structural calculations and is not displayed in the figures shown. Structural coordinates have been deposited in the Protein Databank Bank (PDB: 5IJ4) and NMR chemical shifts in the Biological Magnetic Resonance Bank (BMRB: 30030).

### Sequence Analysis

Protein sequence analysis was performed using the ClustalW2 multiple protein sequence alignment tool [15–16].

### Protein Interaction Studies

Interaction of GST-Cuz1 protein with proteasome or Cdc48 was as previously described [7]. Metal chelation was carried out with o-phenanthroline (5 mM) and EDTA (5 mM) for 30 min at 4°C. Cdc48 was of recombinant bacterial origin (see above). 26S proteasome was affinity purified from yeast expressing *RPN11-TEV-ProA* from the endogenous *RPN11* locus [17].

## Results

### Purification of the Cuz1 AN1 Zinc Finger Domain

**Fig 1:**
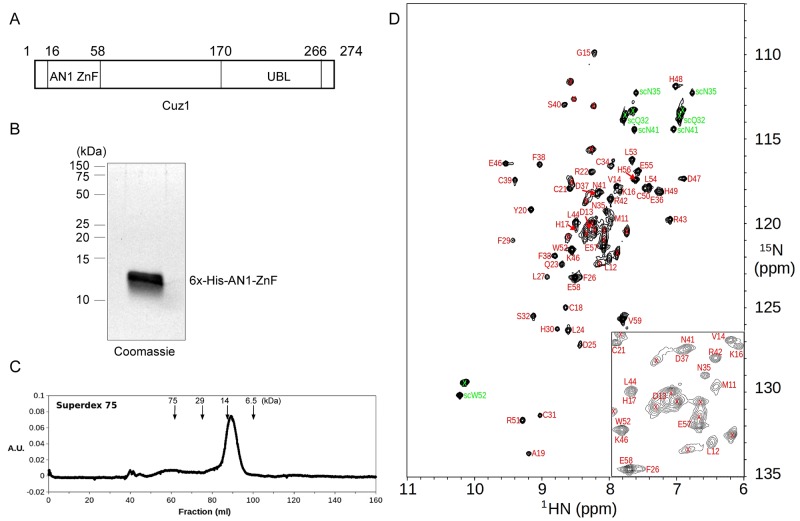
Expression and Purification of the Cuz1 AN1 Zinc Finger Domain. A) Schematic diagram of the Cuz1 protein. UBL, ubiquitin-like domain. B) Purified Cuz1 AN1 ZnF protein (15 μg) as analyzed by SDS-PAGE followed by Coomassie staining. C) Size exclusion chromatography of purified Cuz1 AN1 ZnF protein. Molecular weight size standards are shown for reference. D) ^15^N-HSQC spectrum of the ^15^N-labeled Cuz1 AN1 ZnF domain sample showing assignments of Cuz1 residue resonance peaks. Backbone amide and sidechain peaks are labeled in red and green, respectively, and peaks from the cloning tag are marked with a “x” sign. An expanded view of the central spectral region is shown at the lower right corner.

Cuz1 is one of two AN1 zinc finger proteins in yeast. It is a 274 amino acid protein with two known motifs (Fig 1A). Its AN1 zinc finger domain is present at the N-terminus, and its function is currently unknown. Cuz1 also contains a ubiquitin-like domain at its C-terminus, which is thought to mediate binding to the multifunctional ATPase Cdc48, also known as p97 in higher organisms [6].

To obtain material for structural analysis, we cloned a fragment (residues 11–59) of Cuz1 representing the AN1 zinc finger domain with an N-terminal 6x-histidine tag. We expressed this construct in *E*. *coli*, and purified the protein by nickel-affinity chromatography. After subsequent efforts to improve purity (see Materials and Methods), we obtained the AN1 domain in milligram quantities and purified to near homogeneity (Fig 1B). We evaluated the purified AN1 domain by gel filtration chromatography. The protein eluted as a single peak (Fig 1C), consistent with it behaving as a monomer and without aggregation. In both the SDS-PAGE (Fig 1B) and the gel filtration (Fig 1C) analyses, the protein ran slightly higher than its predicted molecular weight of 9.1 kD. A ^15^N-HSQC (heteronuclear single quantum coherence) spectrum of the purified ^15^N-labeled Cuz1 AN1 domain sample showed well dispersed backbone amide resonance peaks indicating a stably formed structure (Fig 1D). ^15^N-HSQC spectra collected indicate that the Cuz1 ZnF protein sample was stable at 4°C after 6 months, and remain folded at 45°C.

### Structure of the AN1 Zinc Finger Domain

**Fig 2:**
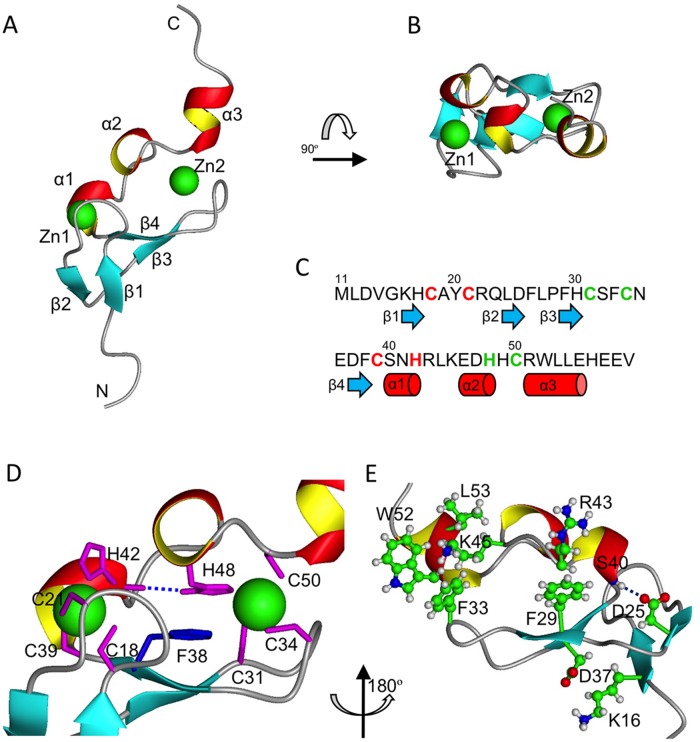
Solution Structure of the AN1 ZnF Showing a Compact Fold. A) Ribbon diagram of the Cuz1 AN1 ZnF domain. Beta strands 1–4 and helices 1–3 are indicated, as well as the two zinc atoms as green spheres. B) Top view (rotated 90° about the X-axis) showing the compactness of the Cuz1 AN1 ZnF domain. Note that the scales are the same for panels A and B. C) Sequence alignment of the Cuz1 AN1 ZnF domain showing secondary structure: blue arrows, beta strands; red cylinders, short alpha helices. Residues coordinating the first zinc cluster are colored in red, and those coordinating the second zinc cluster in green. D) Central position of Phe38 at the core of AN1 ZnF domain between the two zinc chelating clusters. The dotted blue line indicates a hydrogen bond between His48 and His42 which further bridges the two zinc clusters. E) Additional polar and hydrophobic interactions that contribute to the stability of the compact Cuz1 AN1 ZnF domain.

The solution structure of the Cuz1 zinc finger (ZnF) domain was solved by NMR spectroscopy using ^15^N/^13^C labeled protein in Tris buffer (pH 7.5) at 25°C (Fig 1 and S1 Fig; Table 1). The Cuz1 ZnF domain has an AN1-type zinc binding Cys6His2 (CC-CC-CH-HC) motif consisting of two cross-braced Cys3His (CC-CH) and Cys2HisCys (CC-HC) zinc binding clusters (Fig 2A and 2B). The secondary structure of the Cuz1 ZnF consists of two tandem connected β-hairpins (β1:Lys16-His17/β2:Leu24-Asp25 and β3:Phe29-His30/β4:Asp37-Phe38) followed by two short 3/10-helicies (α1:Ser40-His42 and α2:Glu46-His48) and a longer C-terminal α-helix (α3:Arg51-Glu55) (Fig 2C). The overall fold is very compact (Fig 2B) with two zinc atoms bound to the tips of the two hairpin loops. The AN1 ZnF domain is thus analogous to many other two-zinc binding ZnF domains, such as the RING domain and PHD domain [18–19]. As shown in Fig 2D, the first zinc binding cluster is composed of residues Cys18 and Cys21 from within the first loop between the β1 and β2 strands, and Cys39 and His42 from the beginning and end of the α1 helix, respectively. The second zinc binding cluster is composed of residues Cys31 and Cys34 from the second loop between the β3 and β4 strands, His48 from the α2 helix, and Cys50 from the beginning of α3 helix.

**Table 1:** NMR statistics of 15 solution structures of Cuz1 AN1 ZnF*.

**NMR distance and dihedral constraints**	
Distance constraints	
Total NOE	614
Intra-residue	237
Inter-residue	377
Sequential (|i-j| = 1)	155
Medium-range (|i-j| < 4)	78
Long-range (|i-j| > 5)	144
Zinc cluster constraints	60
Hydrogen bond constraints	0
Total dihedral angle restraints	
phi	31
psi	32
**Structure statistics**	
Violations	
Distance constraints (> 0.3Å)	0
Vdw contacts	0
Dihedral angle constraints (> 5°)	0
Ramachandran plot ^†^	
Most favored region	80.6
Allowed region	19.4
Outlier region	0.0
<r.m.s.d.> to mean structure (Å) ^‡^	
Heavy	0.67 +/- 0.13
Backbone	0.13 +/- 0.06

* The 15 structures with lowest target energy functions were selected from 100 conformers calculated by CYANA with 100,000 simulated annealing steps.

^†^ wwPDB (5IJ4) validation results.

^‡^ For residues between Gly15 and Glu55.

The overall tertiary fold of the AN1ZnF can be viewed as a two finger hand holding a tri-helical clamp, locked at both ends by two zinc binding clusters. Additional interactions at the back side of the Cuz1 ZnF domain further stabilize this compact structure (Fig 2E). At the N-terminal end, Lys16 from the β1 strand and Asp37 from the β4 strand are in position to form a salt bridge; a possible hydrogen bond between the Asp25 side-chain from the β2 strand and the backbone carbonyl of Ser40 from the α1 helix, and the hydrophobic interactions between the aromatic ring of Phe29 from the β3 strand and the aliphatic chain of Arg43 also put tight restraints on the α1 helix. At the C-terminal end, the positive charge group of Lys45 from the loop between the α2 and α3 helices is often observed sandwiched between the aromatic rings of Phe33 from the β3/β4 loop and Trp52 from the α3 helix through cation-pi interaction, locking down the C-terminal α3 helix.

**Fig 3:**
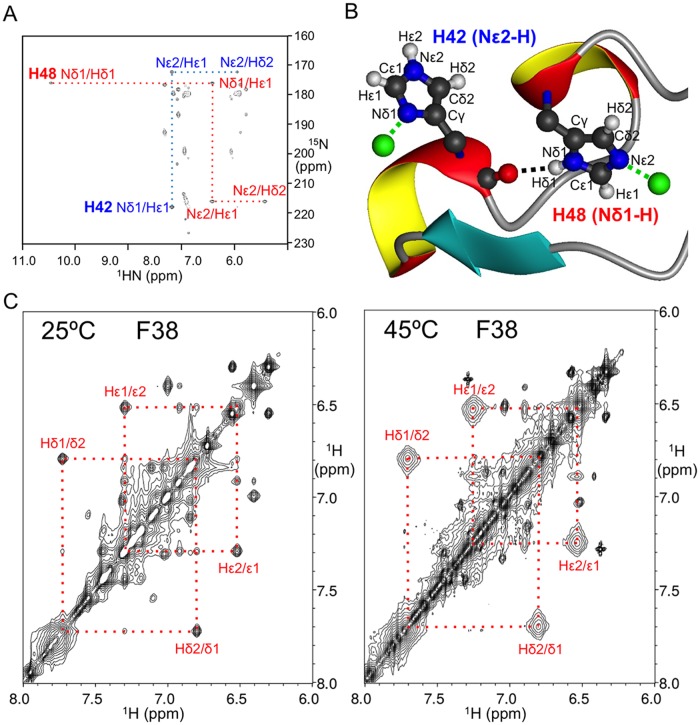
Rigidity of the AN1-ZnF Domain. A) ^15^N-HMQC spectrum of histidine residue side-chain correlations showing the different tautomeric state patterns of zinc-coordinating His42 (blue) and His48 (red). Note the appearance of the His48 Hδ1 resonance peak that is protected from rapid exchanging with H_2_O by a side-chain-to-main-chain hydrogen bond. B) Atomic model of the zinc-coordinating histidine tautomeric states. The black dotted line indicates the side-chain-to-main-chain hydrogen bond involving His42 and His48. C) 2D-NOESY spectra of the Cuz1 ZnF showing chemical exchange cross-peaks between Hδ1/Hδ2 and Hε1/Hε2 spins of Phe38 due to the slow ring-flipping rate, marked by the red dotted lines. Note that the peaks are more broadened at 45°C than at 25°C due to faster chemical exchange, consistent with increasing ring-flipping rates.

The tautomeric states of the zinc-ligated histidine residues in the Cuz1 ZnF domain were determined by acquiring a ^15^N-HMQC (Hetero-nuclear Multiple Quantum Coherence) spectrum (Fig 3A) tuned to the weaker ^2^JHN coupling constants [20–21]. The His42 imidazole ring is in the “HIST” tautomeric state where the Nε2 is protonated and Nδ1 is coordinating the zinc atom in the first zinc-binding cluster; His48 is in the more regular tautomeric state where the Nδ1 is protonated and Nε2 is coordinating the zinc atom in the second zinc-binding cluster (Fig 3B). Interestingly, the same ^15^N-HMQC spectrum showed a His48 Nδ1-Hδ1 cross peak which is usually invisible due to rapid exchanging with the solvent water. Close inspection of NOE constraints networks revealed a hydrogen bond between the His48 side-chain Nδ1-attached Hδ1 and the His42 backbone carbonyl oxygen (Figs 2D and 3A and 3B). This results in significantly reduced solvent exchange and a large down-field shift of the Hδ1 resonance for His48. This unique structural feature bridges the two zinc-binding clusters, the implications of which are discussed below.

### A Conserved Phenylalanine Residue Stabilizes the Zinc-Binding Domains

A phenylalanine at position 38 on the β4 strand is found at the center of the Cuz1 ZnF domain, confined between the two zinc binding clusters (Fig 2D). Its aromatic ring is sandwiched between the polypeptide backbone of the second β-hairpin (β3/β4) and the imidazole ring of His48, which, as mentioned above, is fixated by a side-chain hydrogen bond to the backbone carbonyl oxygen atom of His42. Thus, Phe38 is locked in the center of Cuz1 ZnF domain with little space to move or rotate. Indeed, chemical exchange cross peaks were observed between Hδ1/Hδ2 and Hε1/Hε2 aromatic atoms in the 2D NOESY experiments at temperatures ranging from 25°C to 45°C (Fig 3C). These exchange peaks were clearly absent in a 2D DQF-COSY spectrum as expected. In most cases, rapid aromatic ring flipping rates would normally result in single degenerate Hδ and Hε resonance peaks. The slow 180 degree aromatic ring flipping is uncommon, but has been observed in some prior structures, such as BPTI, cytochrome c, and HPr proteins [22–24]. To prevent chemical exchange peaks from being misinterpreted as erroneous NOE distance constraints, whenever cross peaks were observed in the 2D NOESY spectrum between another atom and both Hδ1/Hδ2, or Hε1/Hε2 atom pairs, the weaker cross peaks were disregarded.

**Fig 4:**
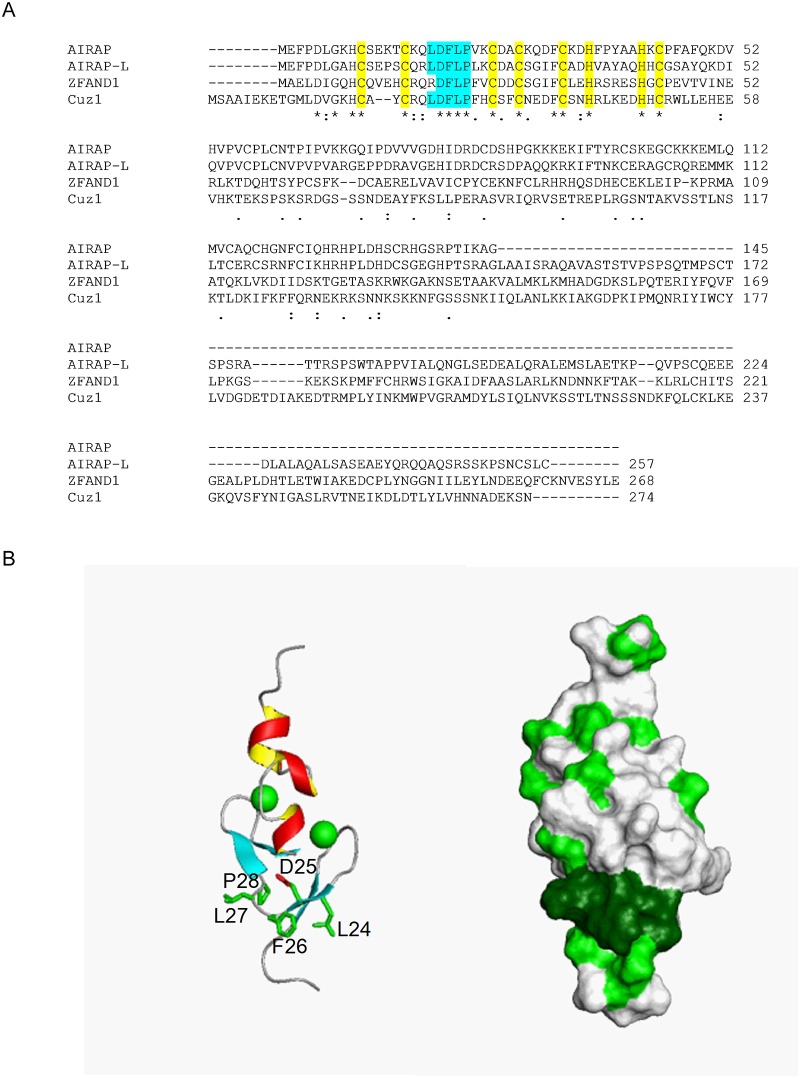
Identification of a Conserved LDFLP Sequence in the Cuz1 AN1 ZnF. A) Sequence alignment of full-length Cuz1 with three human AN1 proteins: AIRAP (Zfand2A), AIRAP-L (Zfand2B), and Zfand1. Zinc chelating residues are highlighted in yellow. The LDFLP motif is highlighted in cyan. Asterisks, identical residues; double dots, highly similar residues; single dots, similar residues. B) Position of the LDFLP motif within the AN1 ZnF. The left panel shows the largely surface exposed LDFLP residues with their side chains. The right panel indicates hydrophobicity in green. Note the hydrophobic patch (in dark green) associated with the LDFLP motif, whereas the remainder of the structure shows a largely unremarkable hydrophobicity distribution.

The position and properties of Phe38 suggested a crucial role in AN1 structure and function. We therefore examined the evolutionary conservation of this residue by sequence analysis. Indeed, a phenylalanine residue preceding the penultimate chelating cysteine residue was found in both yeast AN1 type ZnF domain proteins (Cuz1 and Tmc1), and in six of seven human AN1 proteins (ZFand1, 2A, 2B, 3, 4, and 5; Fig 4A and S2 and S3 Figs). In the sole exception, Zfand6, the corresponding residue is nevertheless the chemically similar amino acid tyrosine (S2 Fig).

### Cuz1 Contains a Conserved Surface Exposed LDFLP Motif

We searched for sequence similarity between Cuz1 and the seven human AN1 proteins described above. The AN1 domain was highly conserved within all of these proteins. Zfand1 has been proposed to represent Cuz1's closest ortholog by virtue of the additional presence in both proteins of a C-terminal ubiquitin-like domain (Ubl) [6]. In addition to Zfand1, we identified additional evidence linking Cuz1 to the related human proteins Zfand2A and Zfand2B, which are also known as AIRAP and AIRAP-like protein (AIRAP-L), respectively. In addition to the zinc chelating residues, there is a second area of absolute evolutionary conservation between Cuz1, AIRAP, and AIRAP-L. This relates to a five residue motif—LDFLP—that occurs after the first cysteine dyad of the zinc finger (Fig 4A). This motif is also highly, although not perfectly, conserved in Zfand1 where the corresponding residues are RDFLP. By contrast, this LDFLP motif is not present in the other yeast AN1 protein, Tmc1, or in any of the remaining four human AN1 proteins (S2 and S3 Figs). There are additional functional similarities between Cuz1, AIRAP, and AIRAP-L which suggest that they are related. All three of these proteins are induced by the presence of trivalent arsenic, hence the name AIRAP for *a*rsenite *i*nducible *R*NA-*a*ssociated *p*rotein [25–26]. Second, all three proteins are known to bind the proteasome, which is the central protease of the ubiquitin-proteasome system [25, 27, 6–7]. Third, both Cuz1 and AIRAP-L interact with Cdc48 (also known as p97 in higher organisms), which is a multifunctional ATPase which functions upstream of the proteasome in the UPS [28, 6–7]. It is unknown whether AIRAP also shares this property. Zfand1 has not been characterized experimentally. Thus, the LDFLP motif appears to define a sub-family of AN1 zinc finger proteins that includes Cuz1 in yeast, and AIRAP, AIRAP-L, and Zfand1 in higher organisms.

Given the evolutionary conservation of the LDFLP motif, we turned our attention to its place within the AN1 domain. In contrast to the other evolutionarily conserved residues within the AN1 domain, the LDFLP residues were not involved in chelating zinc ions. Among these five residues, Asp25 and Pro28 might play some structural role, as the sidechain of Asp25 in the β2 strand could potentially form a hydrogen bond with the backbone amide of Ser40 at the beginning of the α1 helix, while Pro28 may help rigidify the β3/β4 loop. However, the three prominent hydrophobic residues Leu24, Phe26, and Leu27 are not involved in any clustering to stabilize the Cuz1 ZnF, but rather were largely surface exposed (Fig 4B). This hydrophobic surface patch is in contrast to the rest of the ZnF molecular surface which lacks any other significant hydrophobicity or charge distribution patterns (Fig 4B).

### The LDFLP Motif Is Not Required for Cdc48 or Proteasome Binding

**Fig 5:**
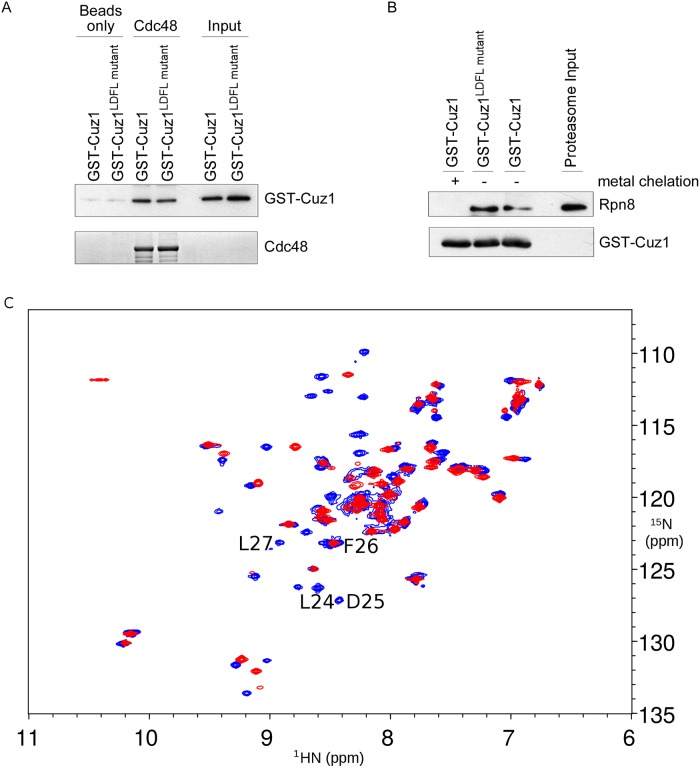
The LDFLP Motif Appears Dispensable for Cdc48- and Proteasome-Binding. A) Binding of GST-tagged Cuz1 or the Cuz1^LDFL→AAAA^ mutant to 12xHis-Sumo-tagged Cdc48 or to beads alone. Binding reactions were visualized by SDS-PAGE followed by immunoblot with anti-Cuz1 antibody (upper panel). Cdc48 levels were analyzed by SDS-PAGE followed by Coomassie staining (lower panel). Input levels of GST-Cuz1 species were visualized to ensure that equivalent amounts of protein were added to the binding assay, and to confirm the assignment of the bands attributed to bound GST-Cuz1. B) Binding of purified proteasome to GST-tagged Cuz1 or the Cuz1^LDFL→AAAA^ mutant, as visualized by SDS-PAGE followed by immunoblot. Upper panel, anti-proteasome subunit Rpn8. Lower panel, anti-Cuz1 immunoblot. Metal chelated GST-Cuz1 serves as a negative control. C) Superimposed ^15^N-HSQC spectra of the AN1 ZnF domains from wild-type Cuz1 (blue) and Cuz1^LDFL→AAAA^ mutant (red). The resonance peaks from mutated residues L24, D25, and L27 are clearly missing (F26 overlaps with another peak).

Cuz1 has two known biochemical functions: it binds Cdc48 and the proteasome [6–7]. We sought to determine whether the LDFLP motif was required for either of these functions. Binding to Cdc48 has been previously localized to Cuz1's C-terminal Ubl domain [6], making any involvement by the AN1 domain or the LDFLP motif unlikely. We tested this directly using purified recombinant full-length Cuz1 and Cdc48 proteins. Wild-type Cuz1 showed strong binding to Cdc48, as previously reported (Fig 5A; [6–7]). We introduced the LDFL→AAAA mutation into the Cuz1 bacterial expression plasmid, and found that the purified mutant protein retained its ability to interact with Cdc48 (Fig 5A).

Cuz1 also interacts with the proteasome, although the precise residues within Cuz1 responsible for this interaction are not known [6–7]. Previous work suggested that a central fragment of Cuz1 might mediate proteasome binding. Although the AN1 domain was not sufficient, binding required both the AN1 domain and a 93 residue fragment immediately C-terminal to the AN1 domain; the C-terminal Ubl domain was not required [6]. Using recombinant purified Cuz1 and intact 26S proteasomes purified from yeast, we found that wild-type Cuz1 and the proteasome interacted, as previously reported (Fig 5B; [6–7]). Interestingly, this interaction was abrogated by zinc chelation, consistent with the previous proposal that the AN1 domain was necessary for this interaction [6]. By contrast, the Cuz1 LDFL mutant retained its ability to bind proteasome (Fig 5B). This result indicates that the LDFLP motif is not required for proteasome binding. Furthermore, this result suggests that LDFL mutant protein retains its zinc binding capacity, consistent with the structural data. Thus, the function of LDFLP motif cannot be accounted for by proteasome or Cdc48 interaction.

Finally, we directly tested whether this motif was required for the structural integrity of the folded ZnF domain. We introduced the LDFL→AAAA mutation into the original AN1 ZnF construct used for structural analysis, and purified the mutant protein. The mutated protein remained folded, as indicated by conservation of a large number of peak positions in the ^15^N-HSQC spectrum (Fig 5C).

## Discussion

We report here for the first time a structural analysis of an AN1 ZnF protein. The AN1 domain represents a compact fold coordinating two zinc ions with six conserved cysteine and two conserved histidine residues. A common feature of the AN1 domain is the presence of an evolutionarily conserved phenylalanine residue situated in between the two zinc chelating clusters, which appears to impart rigidity and stability to the AN1 domain. This is similar to a conserved phenylalanine residue, albeit less tightly packed, in the core of the classical zinc finger [29–30]. The top 8 structure matches for the Cuz1 ZnF domain from a DALI database search are all AN1 domains (e.g. PDB: 1WFE) from the RIKEN Structural Genomic/Proteomics Initiative; however, none of these structures has better defined secondary structure than that presented here (S4 Fig), and no further analysis or characterization of any of these structures were available. The AN1 family of proteins is broad and evolutionarily conserved. In contrast to yeast where there only two such proteins, humans have at least 7 AN1 domain proteins (Zfand1, 2A, 2B, 3–6), although there is limited functional characterization for many of these proteins. We expect, based on the strong evolutionary conservation of the AN1 domain, that the structural data presented here will be broadly applicable to most if not all AN1 ZnF proteins, and may serve as a basis for future mutational and functional analyses of these proteins.

A unique feature of the Cuz1 AN1 ZnF is the presence of a second highly conserved motif, the LDFLP motif, which is located in the connecting loop between the connected β-hairpins. This motif is not present in the other yeast AN1 protein, Tmc1, indicating that it is not an invariable feature of the AN1 domain or necessary for its overall structure. Although this motif might participate in possible hydrogen bond formation, its elimination does not disrupt proper domain folding. Moreover, it is largely surface exposed, particularly with respect to the hydrophobic character contributed by the leucine and phenylalanine residues. This surface exposed hydrophobicity is intriguing, particularly given that the remainder of the AN1 domain shows an otherwise unremarkable surface charge distribution. The potential functional importance of the LDFLP motif is emphasized by its absolute conservation in the human proteins AIRAP and AIRAP-L, and very strong conservation in a third human protein, Zfand1. We propose that the LDFLP motif defines a subfamily of AN1 ZnF proteins that are likely to share common cellular functions.

Two biochemical functions of Cuz1 are known. Cuz1 interacts with the multifunctional ATPase Cdc48; this interaction was previously mapped to Cuz1's C-terminus [6]. Cuz1 also interacts with the proteasome [6–7]. In this case, the ZnF was necessary but not sufficient for proteasome binding [6], a finding further supported by the zinc-dependency of proteasome binding shown here (Fig 5B). However, mutation of the LDFLP motif had no apparent effect on either of these interactions. Binding to ubiquitin has been proposed as a third potential function of Cuz1 [7], although this has remained somewhat uncertain since a prior study failed to detect direct ubiquitin binding [6]. We failed to detect any spectral shifts in the Cuz1 AN1 ZnF after addition of unlabeled recombinant 6xHis-tagged monoubiquitin. However, the strength of this negative result remains uncertain, particularly given that, *in vitro*, Cuz1 preferentially bound longer multi-ubiquitin chains, and showed little or no binding to di- and tri-ubiquitin [7]. We are working towards the goal of preparing a longer ubiquitin chain with sufficient scale to test this model further. Thus, assigning the molecular function of the LDFLP motif will be an important goal for future work. Given the structural and chemical properties of the LDFLP motif, we favor that it mediates an important protein-protein interaction. In this regard, it is worth noting that within the broader AN1 family, the length and amino acid composition of the region in between the two β-hairpins, i.e. the region analogous to the LDFLP motif in Cuz1, is highly variable. This feature of the AN1 domain may allow for versatility and diversity in mediating protein-specific functions.

## Supporting Information

S1 Fig Solution structures of Cuz1-ZnF Domain. Stereo images (side-by-side) of backbone superimposed NMR solution structure ensemble containing 15 conformers. The average backbone (red lines) RMSD is 0.13 Å in the twin zinc-finger core. Side-chains of cysteine and histidine residues involved in zinc atom (green spheres) coordination are shown with blue lines.
(TIF)

S2 Fig Sequence alignment of Cuz1 (S. cerevisiae) and the human proteins Zfand3-6. Due to the length of Zfand4, residues 1–459 were omitted. Note that the LDFLP motif in Cuz1 is not shared with any of these proteins. Asterisks, identical residues; double dots, highly similar residues; single dots, similar residues.
(TIF)

S3 Fig Sequence alignment of the two yeast AN1 proteins, Cuz1 and Tmc1. Note that the LDFLP motif in Cuz1 is not shared with Tmc1. Asterisks, identical residues; double dots, highly similar residues; single dots, similar residues.
(TIF)

S4 Fig Comparison with other AN1-ZnF Domains. A) Ribbon representation of Cuz1-ZnF (PDB: 5IJ4) and the second AN1-ZnF domain of mouse Zfand1 (PDB: 1WFE) superimposed by backbone (with an average RMSD of 1.4 Å). B) Ribbon representation of Cuz1-ZnF and the first AN1-ZnF domain of mouse Zfand1 (PDB: 1WYS) superimposed by backbone (with an average RMSD of 2.0 Å). The secondary structure elements of Cuz1-ZnF are colored in red and yellow, while those from mouse Zfand1 in blue and cyan. The DALI Z-score between Cuz1-ZnF and 1WFE is 4.3 with 30% identical sequence, and the Z-score between Cuz1-ZnF and 1WYS is 2.7 with 49% identical sequence.
(TIF)
