# Supplementary figures and images for: Solution Structure of the Cuz1 AN1 Zinc Finger Domain: An Exposed LDFLP Motif Defines a Subfamily of AN1 Proteins
Source: PLoS One. 2016 Sep 23;11(9):e0163660. doi: 10.1371/journal.pone.0163660 (PMC5035049; doi:10.1371/journal.pone.0163660)

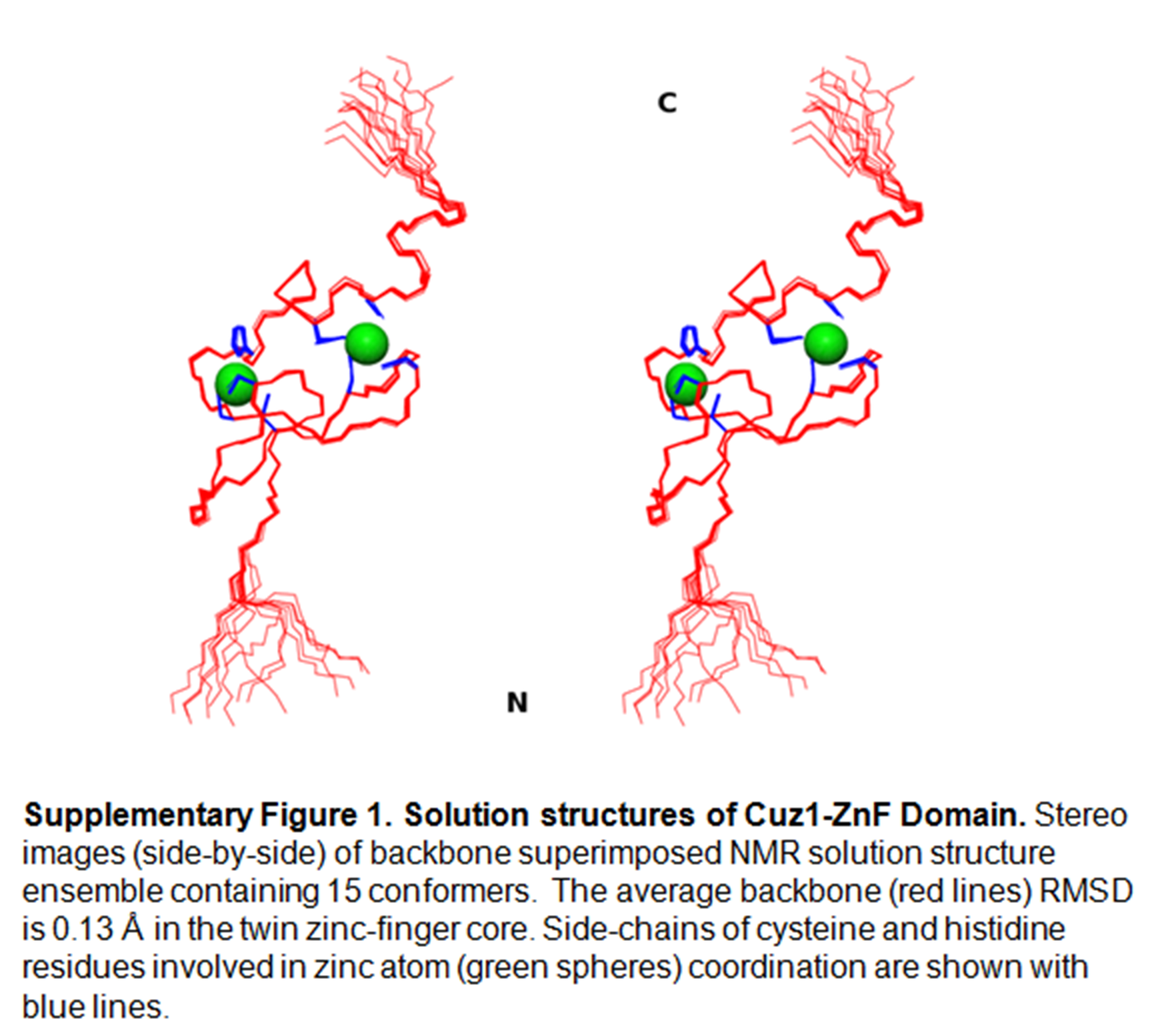

Supplement: S1 Fig — Stereo images (side-by-side) of backbone superimposed NMR solution structure ensemble containing 15 conformers. The average backbone (red lines) RMSD is 0.13 Å in the twin zinc-finger core. Side-chains of cysteine and histidine residues involved in zinc atom (green spheres) coordination are shown with blue lines. (TIF) [file pone.0163660.s001.tif]

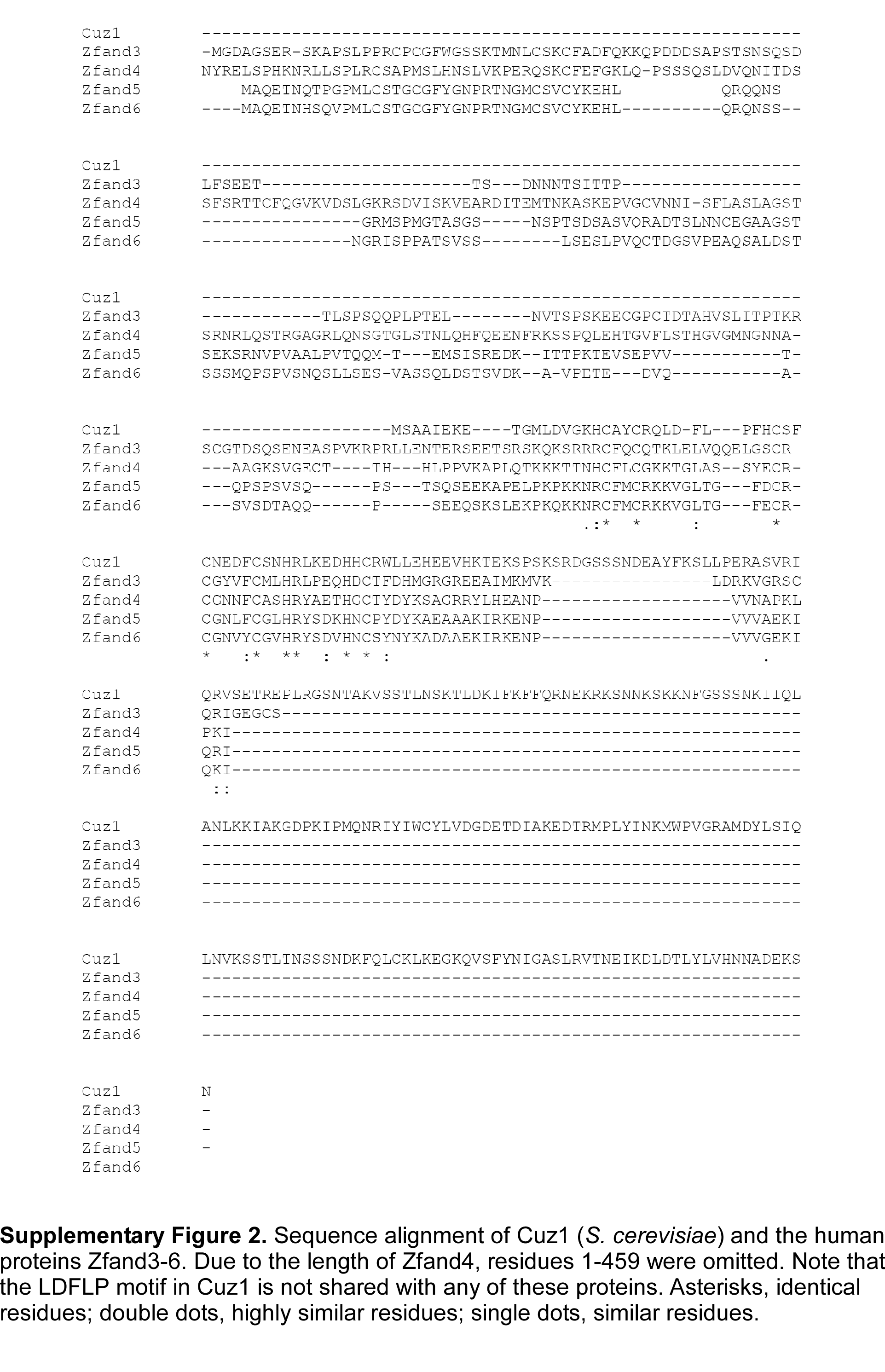

Supplement: S2 Fig — Due to the length of Zfand4, residues 1–459 were omitted. Note that the LDFLP motif in Cuz1 is not shared with any of these proteins. Asterisks, identical residues; double dots, highly similar residues; single dots, similar residues. (TIF) [file pone.0163660.s002.tif]

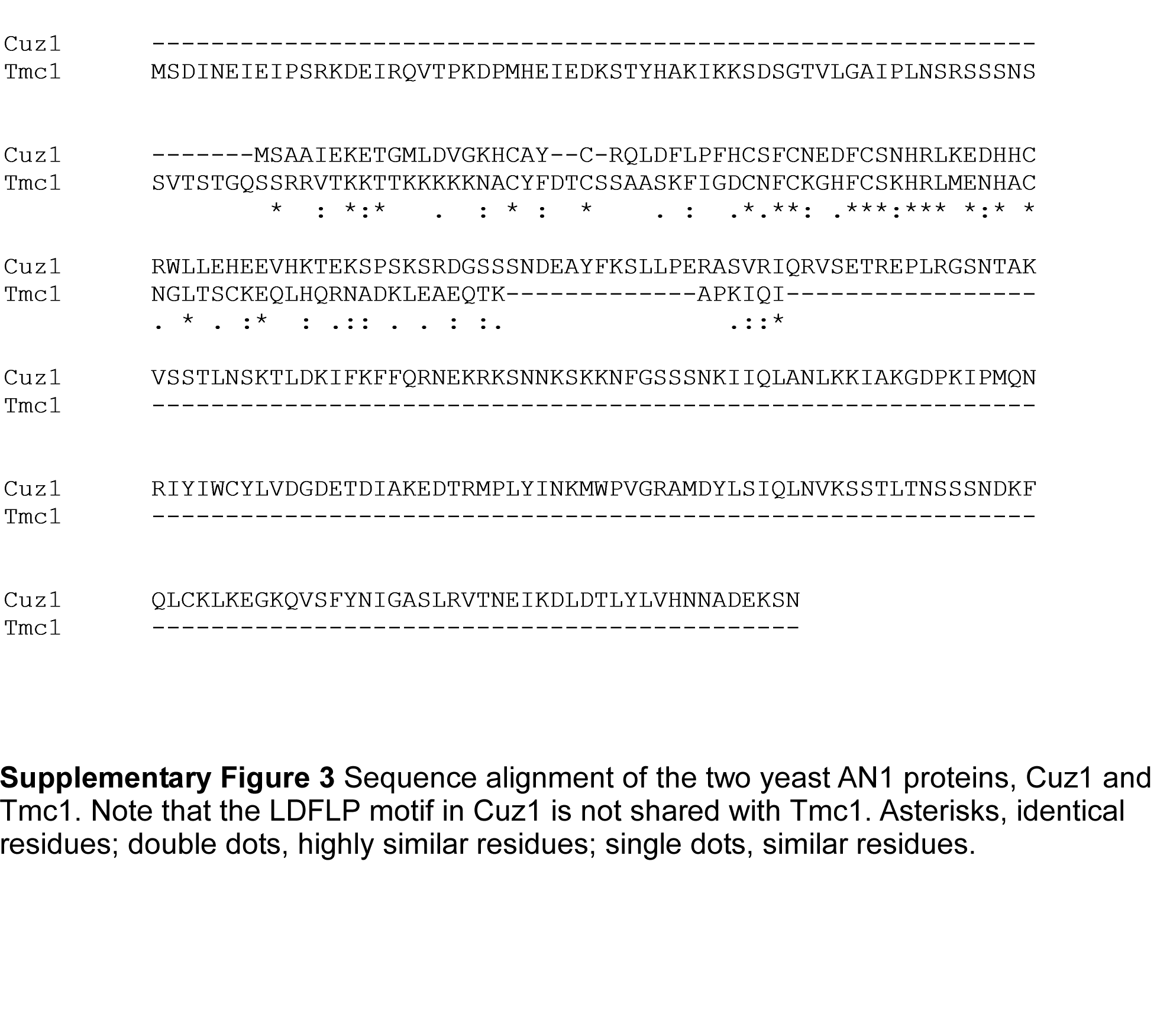

Supplement: S3 Fig — Note that the LDFLP motif in Cuz1 is not shared with Tmc1. Asterisks, identical residues; double dots, highly similar residues; single dots, similar residues. (TIF) [file pone.0163660.s003.tif]

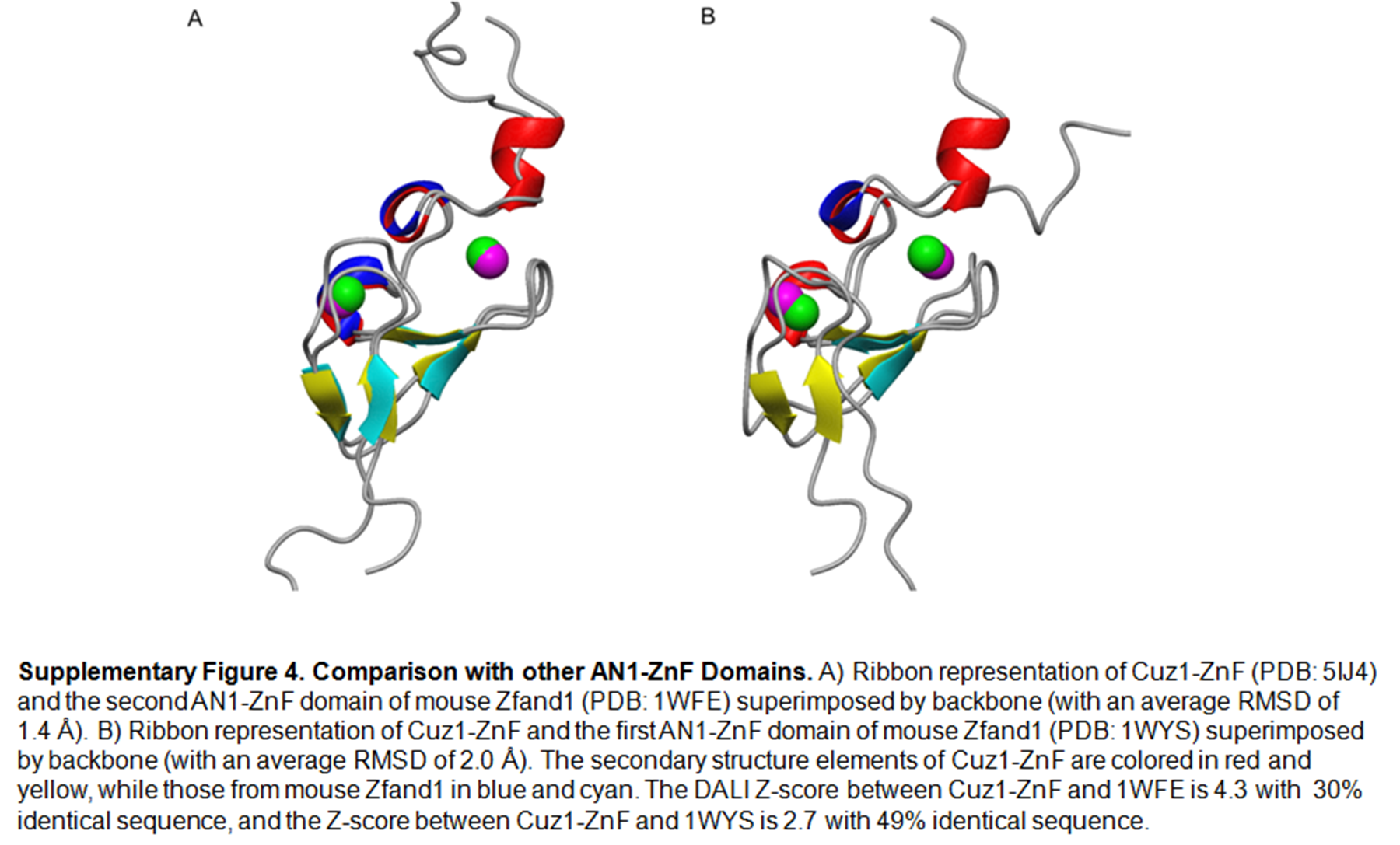

Supplement: S4 Fig — A) Ribbon representation of Cuz1-ZnF (PDB: 5IJ4) and the second AN1-ZnF domain of mouse Zfand1 (PDB: 1WFE) superimposed by backbone (with an average RMSD of 1.4 Å). B) Ribbon representation of Cuz1-ZnF and the first AN1-ZnF domain of mouse Zfand1 (PDB: 1WYS) superimposed by backbone (with an average RMSD of 2.0 Å). The secondary structure elements of Cuz1-ZnF are colored in red and yellow, while those from mouse Zfand1 in blue and cyan. The DALI Z-score between Cuz1-ZnF and 1WFE is 4.3 with 30% identical sequence, and the Z-score between Cuz1-ZnF and 1WYS is 2.7 with 49% identical sequence. (TIF) [file pone.0163660.s004.tif]
